# The complete plastome sequence of a subtropical tree *Pyrus betulaefolia* (Rosaceae)

**DOI:** 10.1080/23802359.2020.1715889

**Published:** 2020-01-24

**Authors:** Zhanghong Dong, Shaohong Qu, Xianhuang Li, Peng Ye, Cheng Liu, Yaxuan Xin, Peiyao Xin

**Affiliations:** Key Laboratory of Forest Resources Conservation and Utilization in the Southwest Mountains of China Ministry of Education, Southwest Forestry University, Kunming, China

**Keywords:** *Pyrus*, chloroplast, phylogenetic analysis

## Abstract

The genus *Pyrus*, comprising several popular fruit crops worldwide, includes over 30 tree species. Here we determined the complete plastid genome sequence of *Pyrus betulaefolia*. The plastome consists of 160,184 bp, including a pair of inverted repeats (IRs) with a length of 26,384 bp separated by a large single-copy region (LSC) and a small single-copy region (SSC) of 88,121 bp and 19,295 bp, respectively. Further phylogenetic analyze was conducted using 11 complete plastid genomes of Rosaceae with KVM + F + I model, which supports *Pyrus betulaefolia* as a sister to all other eight *Pyrus* taxa with published plastomes.

*Pyrus betulaefolia* Bunge is a subtropical tree that mainly distributed in northern China (http://foc.iplant.cn/). Zong et al. (2013) suggest that its developed root system has a strong ability for *P. betulaefolia* in cold resistance, drought resistance, and saline-alkali tolerance. It was also presumably considered as one of the ancient species of the genus *Pyrus* (Rubtsov 1944; Zheng et al. 2011). *P. betulaefolia* has good grafting compatibility with other species of *Pyrus* genus, which is mainly used as the rootstock of all kinds of cultivated pear and is also an important parent in pear dwarfing rootstock and resistance breeding (Okubo and Sakuratani 2000; Robbani et al. 2006). For a better understanding of the relationships of *P. betulaefolia* and other *Pyrus* species, it is necessary to reconstruct a phylogenetic tree based on high-throughput sequencing approaches.

Young leaves of *P. betulaefolia* were collected from the Ruili Botanical Garden (Long. 97.8185 E, Lat. 24.0714N, 1165m) for genomic DNA extraction using the N-Lauroylsarcosine sodium salt method (Wu et al. 2017). The voucher was deposited at the Key Laboratory of Forest Resources Conservation and Utilization in the Southwest Mountains of China Ministry of Education, Southwest Forestry University (Accession Number: SWFU–SY36748). The whole plastome was sequenced following Zhang et al. (2016), and the long-range PCR was used for next-generation sequencing with 15 pairs of universal primers. The contigs were aligned using the publicly available plastid genome of *P. ussuriensis* (Accession Number: MK172841) (Gil et al. 2019) and annotated in Geneious 8.1.9.

The plastome of *P. betulaefolia* (LAU10003) with a length of 160,184 bp, was the largest of the 25 reported plastome of *Pyrus*, was 27 bp and 1023 bp larger than that of *P. ussuriensis* (160,157 bp, MK172841) and *P. spinosa* (159,161 bp, NC023130). The length of the large single-copy (LSC), inverted repeats (IRs), and small single-copy (SSC) regions of *P. betulaefolia* was 88,121 bp, 26,384 bp, and 19,295 bp, respectively. The overall GC content is 36.5% (LSC, 34.1%; IR, 42.7%; SSC, 30.3%). The *P. betulaefolia* plastid genome encoded a set of 133 genes, of which 88 are protein-coding genes, 8 are rRNA genes, and 37 are tRNA genes.

Furthermore, in order to confirm the evolutionary relationship between *P. betulaefolia* and other species with published plastomes in *Pyrus*, we reconstructed a phylogenetic tree (Figure 1) based on 10 published plastid genome sequences of the Rosaceae. *Malus domestica* (Accession Number: MH595623) was treated as an out-group, aligned by the MAFFT version 11 program (Katoh and Standley 2013). A maximum-likelihood (ML) analysis based on the KVM + F+I model was performed with iqtree version 1.6.7 program using 1000 bootstrap replicates (Nguyen et al. 2015). The ML phylogenetic tree with 68–100% bootstrap values at each node supported that *P. betulaefolia* as a sister to all other eight *Pyrus* taxa with published plastomes.

**Figure 1.**
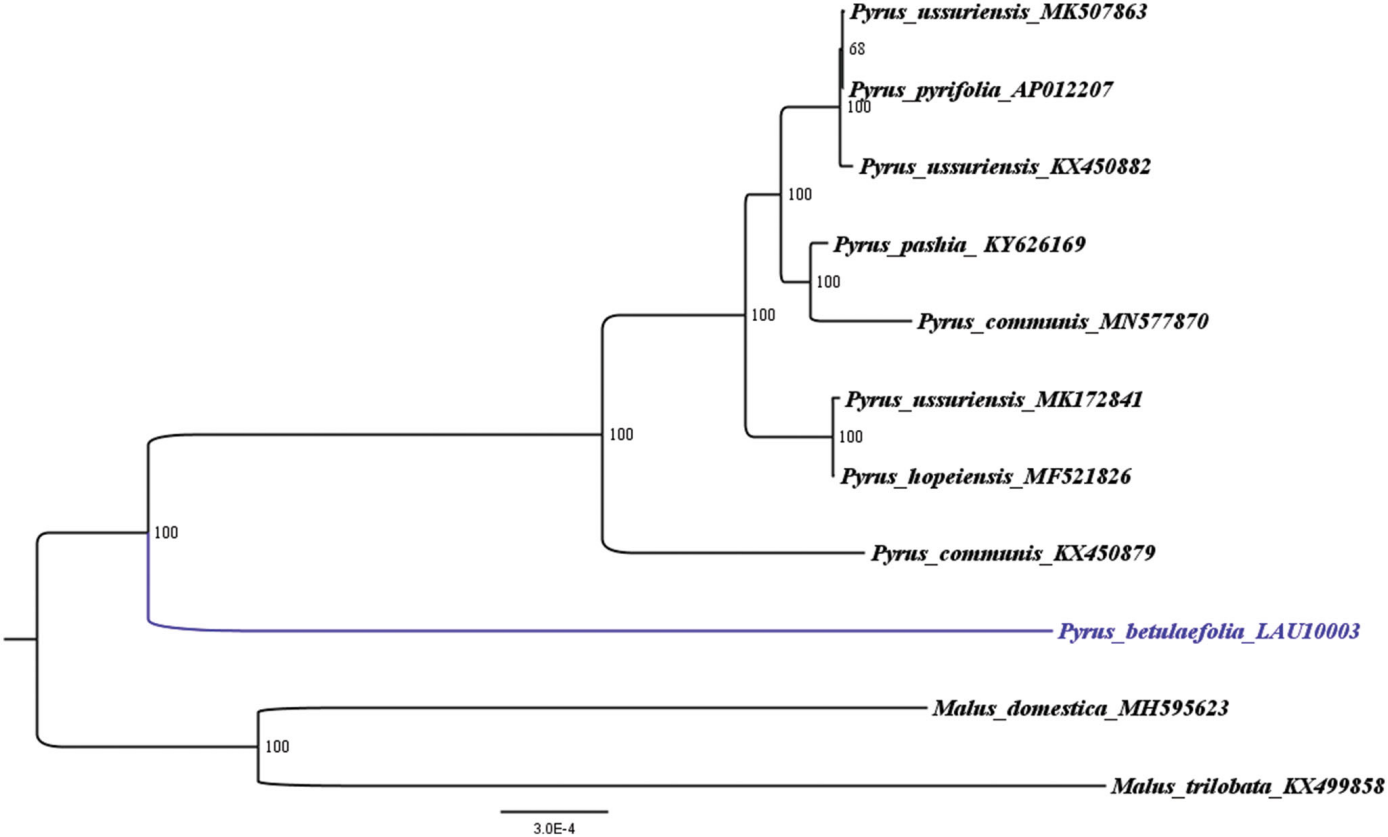
The ML phylogenetic tree for *P. betulaefolia* based on other 10 species (8 in *Pyrus* and 2 in *Malus*) chloroplast genomes.

## Data Availability

The chloroplast data of the *P. betulaefolia* will be submitted to Rosaceae Chloroplast Genome Database (https://lcgdb.wordpress.com). Accession numbers are LAU10003.
